# Supplementary figures and images for: Excitatory projections from the nucleus reuniens to the medial prefrontal cortex modulate pain and depression-like behaviors in mice
Source: PLoS Biol. 2025 May 20;23(5):e3003170. doi: 10.1371/journal.pbio.3003170 (PMC12091829; doi:10.1371/journal.pbio.3003170)

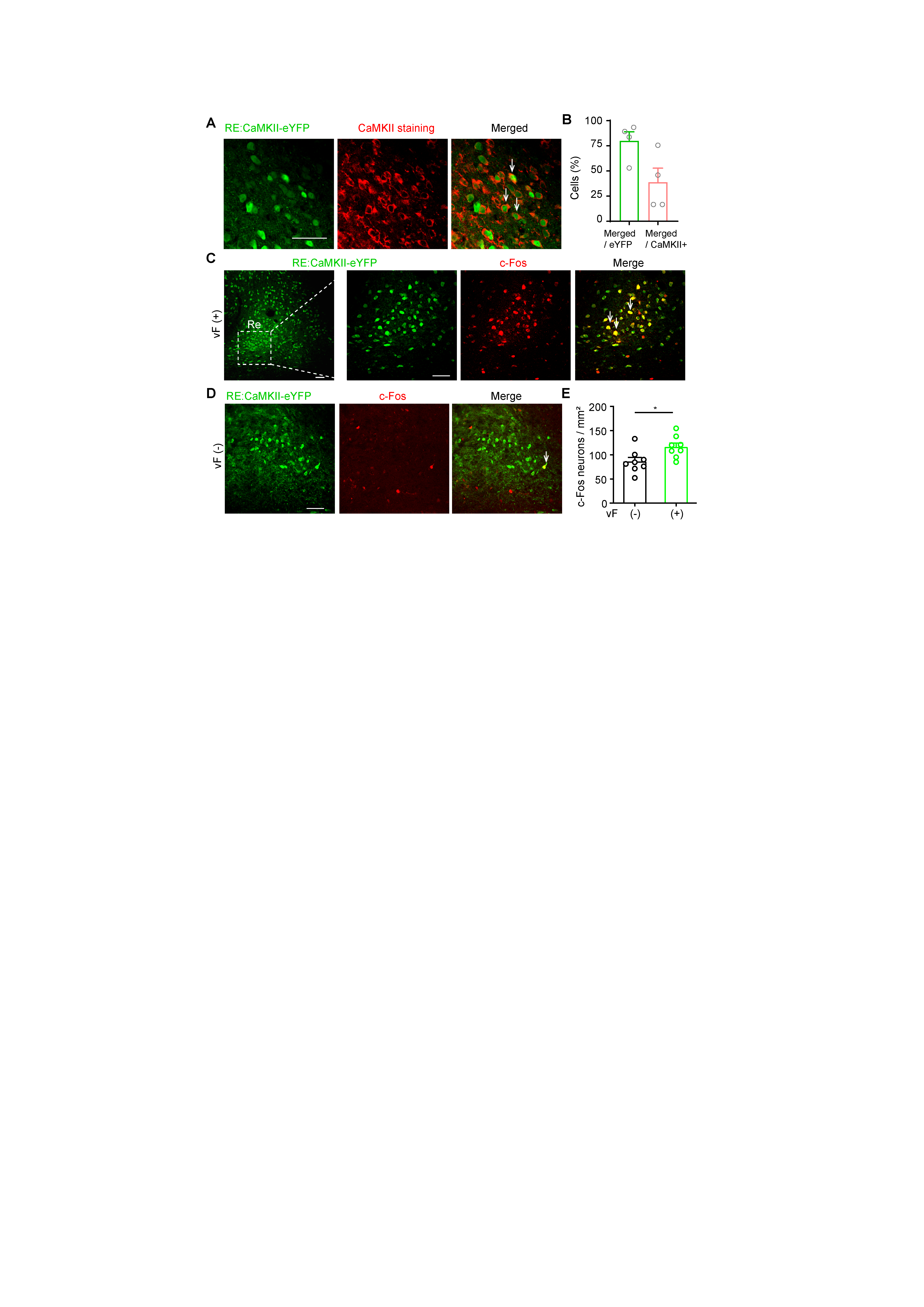

Supplement: S1 Fig — (A, B) Representative images and summary showing that injection of AAV-CaMKII-ChR2-eYFP into the RE transfected eYFP into the RE neurons, most of which were immunofluorescently stained with the CaMKII-antibody (red). n = 4 mice. (C, D) Representative images showing the labeling of RE neurons with AAV-CaMKII-eYFP and c-Fos-antibody-staining (red) in mice whose hind paws received (C, vF(+)) or did not receive (D, vF(−)) stimulation of the 2 g von Frey filament. Arrows indicate overlapping of c-Fos with eYFP. (E) Quantification of percent of eYFP-expressing RE neurons stained with c-Fos-antibody. t = 2.62, P = 0.02, n = 8 mice. Two-tailed unpaired t test. Data are available in S1 Data as a part of Supporting information. vF, von Frey filament. Scale bar: 100 μm. (TIFF) [file pbio.3003170.s001.tiff]

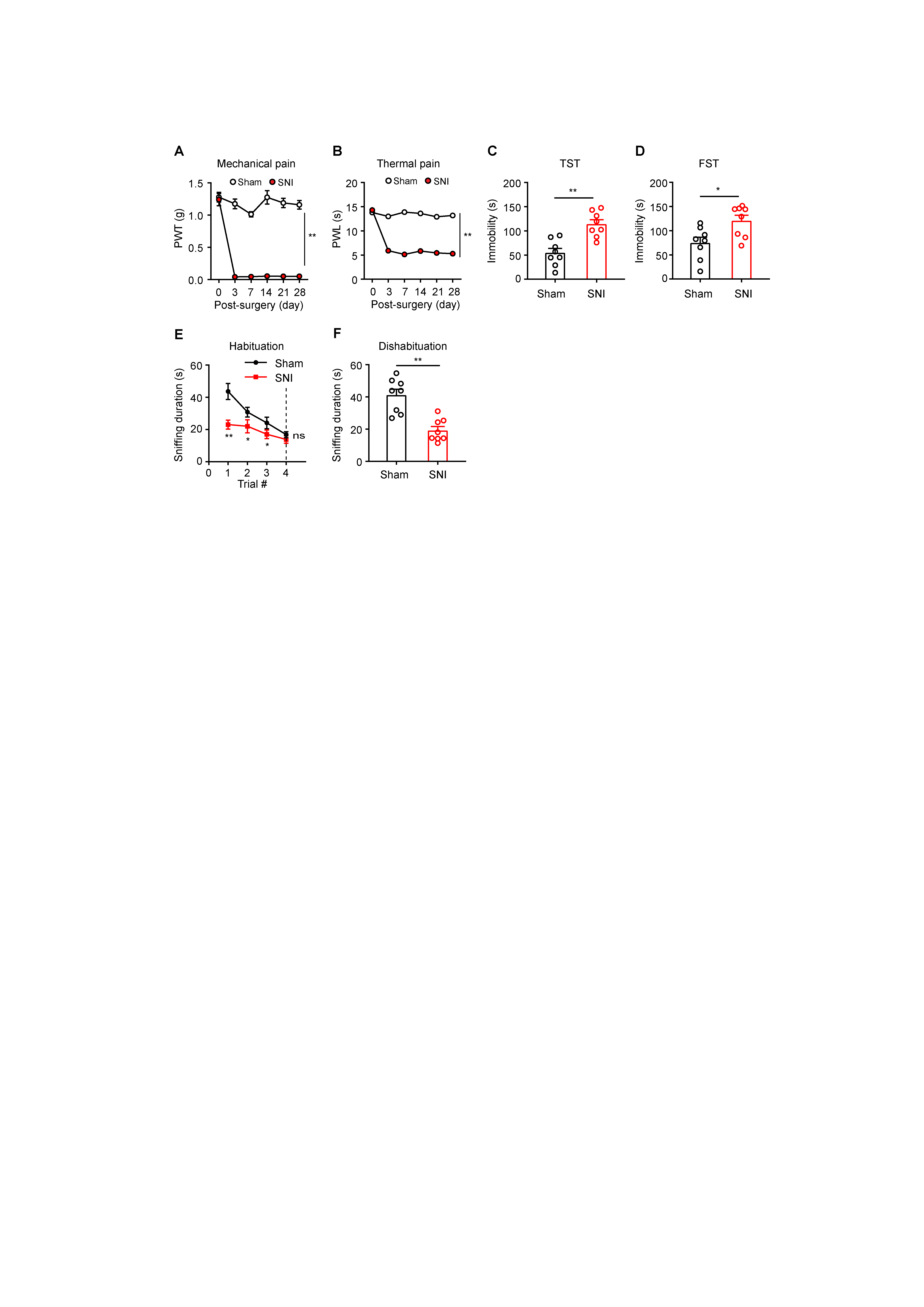

Supplement: S2 Fig — (A, B) Time courses of mechanical withdrawal threshold (PWT) (left panel) and thermal (right panel) withdrawal latency (PWL) after SNI and sham surgery. (A) F(5, 70) = 20.8, P < 0.0001. (B) F(5, 70) = 65.16, P < 0.0001. n = 8 in each group. (C, D) Immobility time in the tail suspension test (TST) (C, t = 2.79, P = 0.01, n = 8 in each group) and the forced swim test (FST) (D, t = 4.5, P = 0.0005, n = 8 in each group). (E, F) Total time spent sniffing the same stimulus mice by sham and SNI mice over 4 trials with 1 h intervals between trials (E, F(1, 18) = 13.26, P = 0.002). (F) Total time that sham and SNI mice spent sniffing the novel stimulus mice. t = 4.92, P = 0.0002. *P < 0.05, **P < 0.01, ns not significant. Two-tailed unpaired t test for (C, D, F); Two-way repeated measures ANOVAs with Tukey’s post-hoc analysis for (A, B, E). Data are available in S1 Data as a part of Supporting information. (TIFF) [file pbio.3003170.s002.tiff]

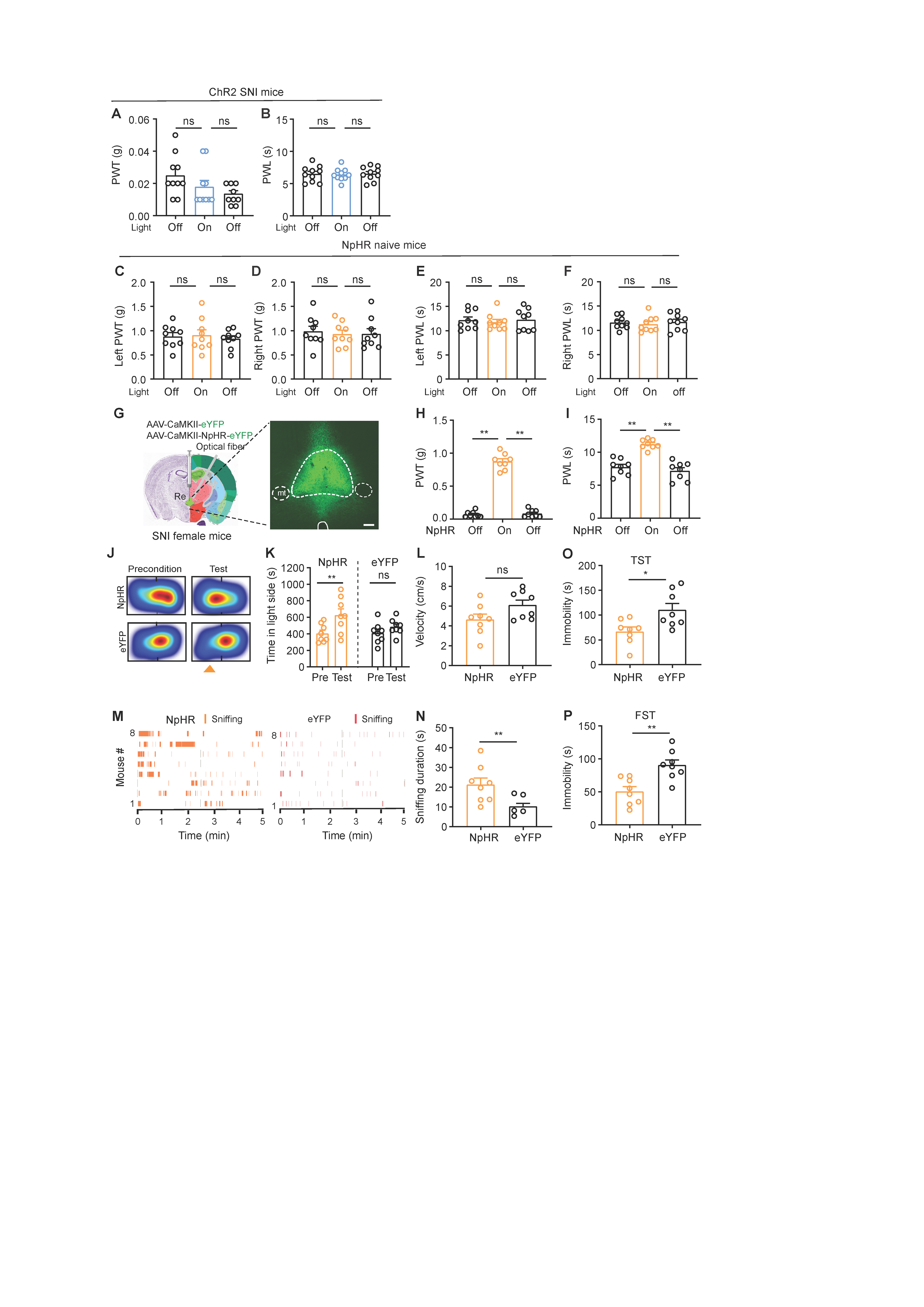

Supplement: S3 Fig — (A, B) Effect of blue light illumination of RE neurons on mechanical PWT (A, F(2, 27) = 2.81, P = 0.08) and thermal PWL (B, F(2, 27) = 0.04, P = 0.96) in ChR2 mice subjected to SNI surgery (n = 10 SNI mice). (C–F) Effect of optogenetic inhibition of RE neurons on mechanical paw withdrawal threshold (PWT) (C, F(2, 24) = 0.19, P = 0.83; D, F(2, 24) = 0.17, P = 0.85; n = 9 each group) and thermal paw withdrawal latency (PWL) (E, F(2, 24) = 0.17, P = 0.85; F, F(2, 24) = 0.23, P = 0.79) on either hind paw of naïve NpHR mice (n = 9). (G) Schematic diagram and representative image of viral expression in the RE for optogenetic inhibition of RE neurons in SNI female mice. Left panel: Nissl (left part of slices) and anatomical annotations (right part of slices) from the Allen Mouse Brain Atlas (mouse.brain-map.org) and Allen Reference Atlas-Mouse Brain (atlas.brain-map.org). (H, I) Effect of NpHR-mediated inhibition of RE neurons on mechanical PWT (F(2, 21) = 25.73, P < 0.0001) and thermal PWL (F(2, 21) = 29.62, P < 0.0001) in SNI mice (n = 8 mice). (J, K) Example heat maps (J) and quantification of time spent (K, Time, F(1, 14) = 14.79, P = 0.0018) in the yellow-light-paired chamber during the preconditioning (Pre) and test sessions for NpHR mice (n = 8) and eYFP mice (n = 8). (L) Velocity of NpHR mice and eYFP mice in light paired-chamber during the test session (t = 1.97, P = 0.069, n = 8). (M, N) Raster plot showing sniffing episode (M) and total time spent sniffing (N, t = 3.02, P = 0.0092) in NpHR (n = 8) and eYFP (n = 8) mice tested during yellow light illumination of the RE. (O, P) Immobility time in the FST (O, t = 2.78, P = 0.015) and TST (P, t = 3.80, P = 0.002) in NpHR (n = 8) and eYFP (n = 8) mice during yellow light illumination of RE neurons. *P < 0.05; **P < 0.01; ns not significant. One-way repeated measures ANOVAs for (A − F, H, I). Two-way ANOVA with Tukey’s post-hoc analysis for (K). Two-tailed unpaired t-tests for (L, N − P). Data are available in S1 Data [file pbio.3003170.s003.tiff]

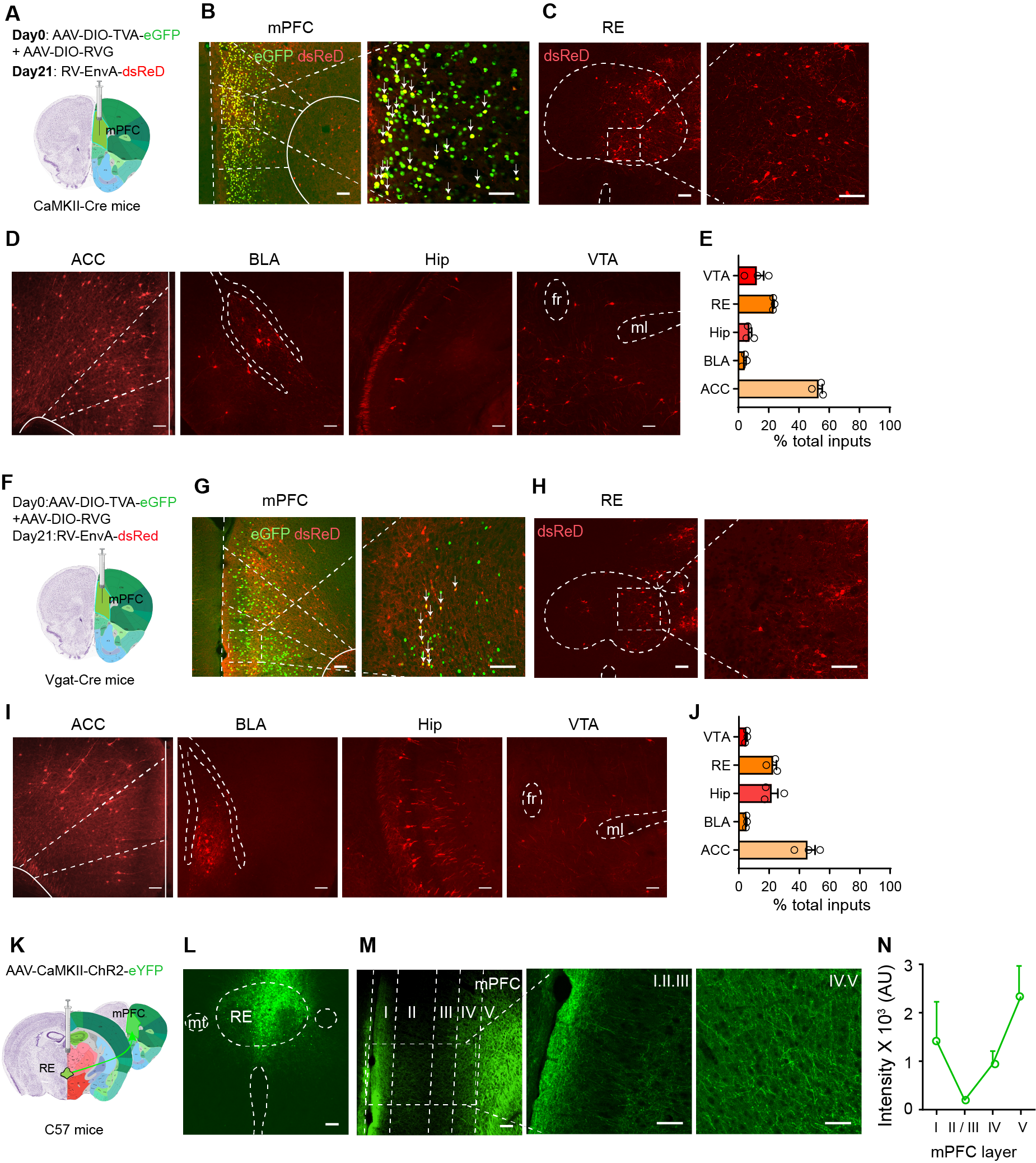

Supplement: S4 Fig — (A) Schematic diagram of the neuronal tracing strategy for probing upstream nuclei of mPFC glutamatergic (Glu) neurons. Nissl (left) and anatomical annotations (right) from the Allen Mouse Brain Atlas (https://mouse.brain-map.org) and Allen Reference Atlas-Mouse Brain (https://atlas.brain-map.org). (B) The injection site and viral expression (right) in the mPFC (Arrows indicate starter cells). (C − E) Representative images and quantification of upstream nuclei of mPFC Glu neurons, including the RE and other brain regions. n = 3 mice. (F) Schematic diagram of the virus tracing strategy for probing the upstream nuclei of mPFC GABAergic neurons. Nissl (left) and anatomical annotations (right) from the Allen Mouse Brain Atlas (https://mouse.brain-map.org) and Allen Reference Atlas-Mouse Brain (https://atlas.brain-map.org). (G) The injected site and viral expression in the mPFC (Arrows indicate starter cells). (H − J) Representative images and quantification of upstream nuclei of mPFC GABAergic neurons, including the RE and other brain regions. n = 3 mice. (K, L) Schematic diagram for virus injection (K) and example image (L) showing that AAV-CaMKII-ChR2-eYFP was injected into the RE. (M, N) Example images (M) and quantification (N) of ChR2-eYFP-labeled fibers in the layers I-V of the mPFC (n = 5 mice). (K) Nissl (left) and anatomical annotations (right) from the Allen Mouse Brain Atlas (mouse.brain-map.org) and Allen Reference Atlas-Mouse Brain (atlas.brain-map.org). (N) The axonal terminals were quantified by pixels of eYFP. Scale bars: 100 μm. Data are available in S1 Data as a part of Supporting information. ACC, anterior cingulate cortex; BLA, basolateral amygdala; fr, fasciculus retroflexus; Hip, hippocampus; ml, medial lemniscus; mPFC, medial prefrontal cortex; RE, nucleus reuniens of the thalamus; VTA, ventral tegmental area. (TIFF) [file pbio.3003170.s004.tiff]

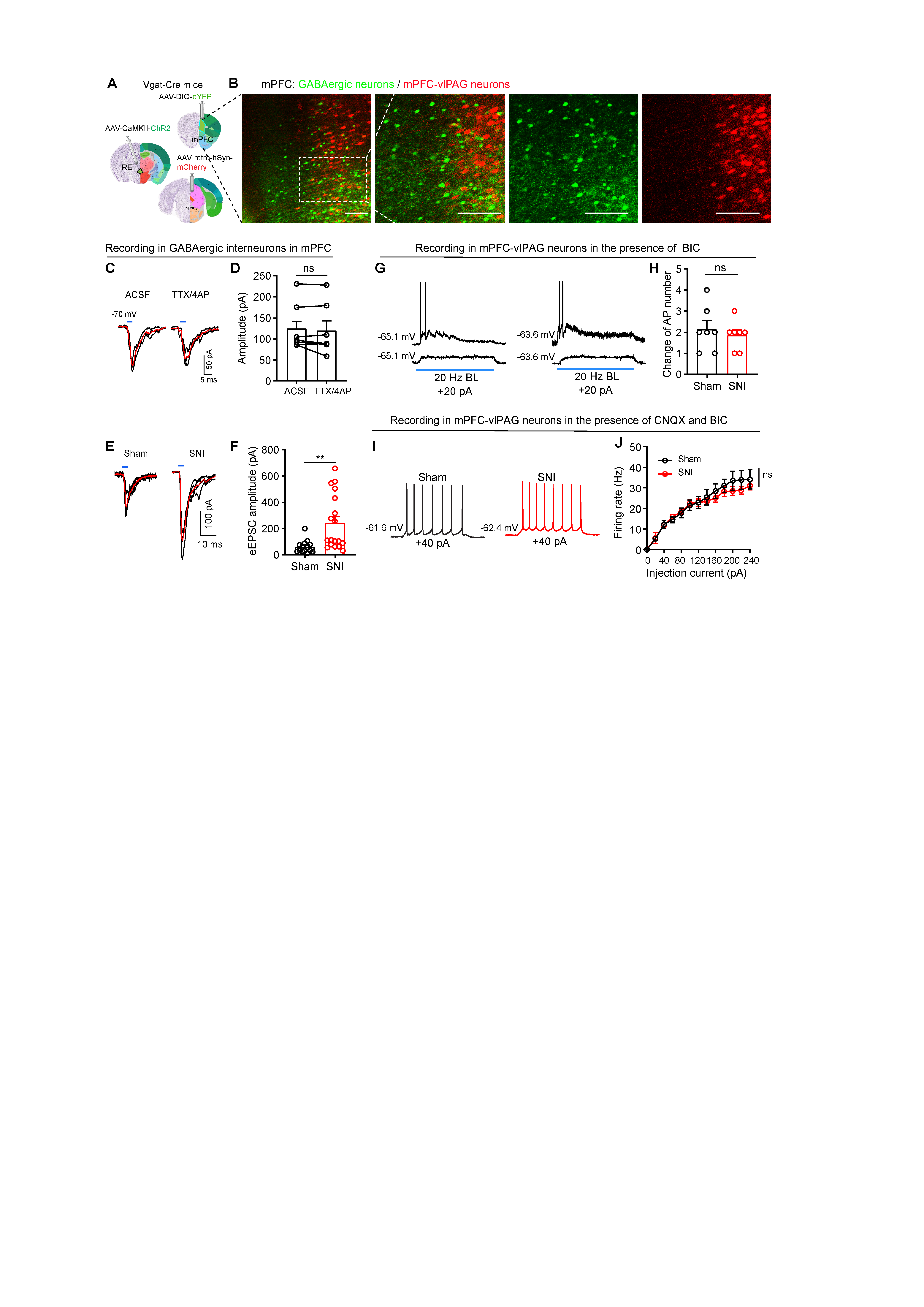

Supplement: S5 Fig — (A) Diagram shows virus injection strategy for labeling mPFC GABA neurons and mPFC-vlPAG projecting neurons and recording their response to optogenetic activation of RE neurons. Nissl (left part of slices) and anatomical annotations (right part of slices) from the Allen Mouse Brain Atlas (https://mouse.brain-map.org) and Allen Reference Atlas-Mouse Brain (https://atlas.brain-map.org). (B) Representative images of labeled neurons in the mPFC. (C) Representative traces of blue light-evoked EPSC (recorded at −70 mV) in eYFP-labeled GABA neurons before and after application of TTX (1 µM) and 4-AP (100 µM). (D) Quantification of amplitude of light-evoked EPSCs on labeled GABA neurons before and during application of TTX and 4-AP. t = 1.13, P = 0.30, n = 7 cells from 4 mice. (E, F) Representative traces and summary of amplitudes of eEPSCs recorded from labeled GABA neurons (−70 mV) receiving RE glutamatergic projections in SNI and sham mice. t = 3.05, P = 0.005, n = 13 − 18 cells from 5 mice in each group. (G, H) Representative traces and summaries of changes in number of firing evoked by 1 s blue light (BL) in mPFC-vlPAG neurons in sham and SNI mice. t = 1, P = 0.36, n = 7 cells each group. (I, J) Example traces and summary of frequencies of firing evoked by depolarizing current injection in mPFC GABA neurons from sham and SNI mice in the presence of 20 μM CNQX and 10 μM bicuculline. F(1, 10) = 0.25, P = 0.63, n = 8 cells from 5 mice in each group. Scale bars: 100 μm. BL, blue light. *P < 0.05, **P < 0.01, ns not significant. Two-tailed paired t-tests for (D). Two-tailed unpaired t test for (F, H). Two-way repeated measures ANOVA with Tukey’s post-hoc analysis for (J). Data are available in S1 Data as a part of Supporting information. (TIFF) [file pbio.3003170.s005.tiff]

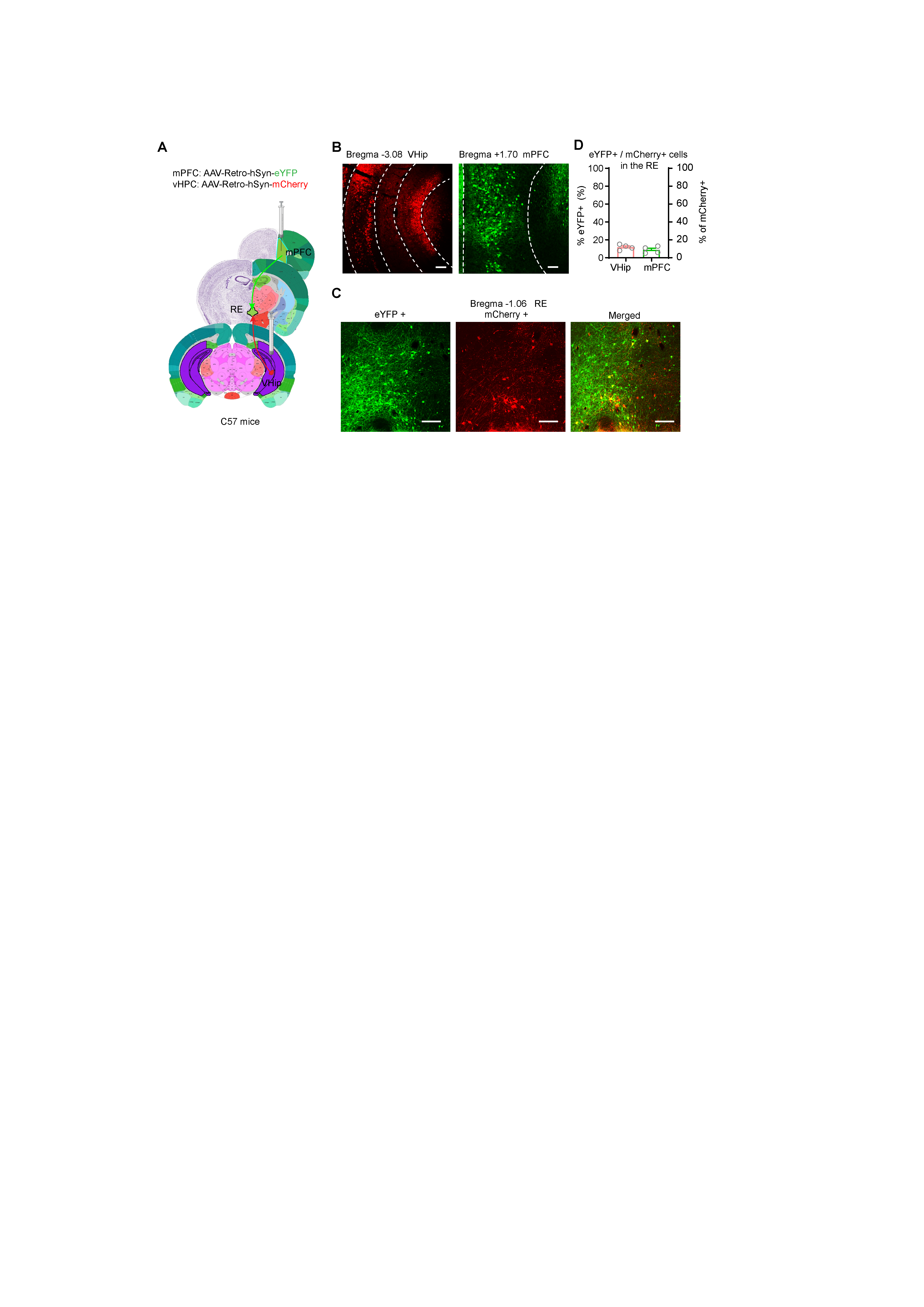

Supplement: S6 Fig — (A) Schematic diagram for labeling of upstream nuclei of the ventral hippocampus (VHip) and medial prefrontal cortex (mPFC). Nissl (left part of slices) and anatomical annotations (right part of slices) from the Allen Mouse Brain Atlas (https://mouse.brain-map.org) and Allen Reference Atlas-Mouse Brain (https://atlas.brain-map.org). (B) Representative coronal sections showing retrograde virus injection into the VHip (AAV retro-hSyn-mCherry) and mPFC (AAV-retro-hSyn-eYFP). (C, D) Example images (C) and summary (D) of RE-VHip (red) and RE-mPFC (green) neurons (n = 4 mice). Data are available in S1 Data as a part of Supporting information. Scale bars: 100 µm. (TIFF) [file pbio.3003170.s006.tiff]

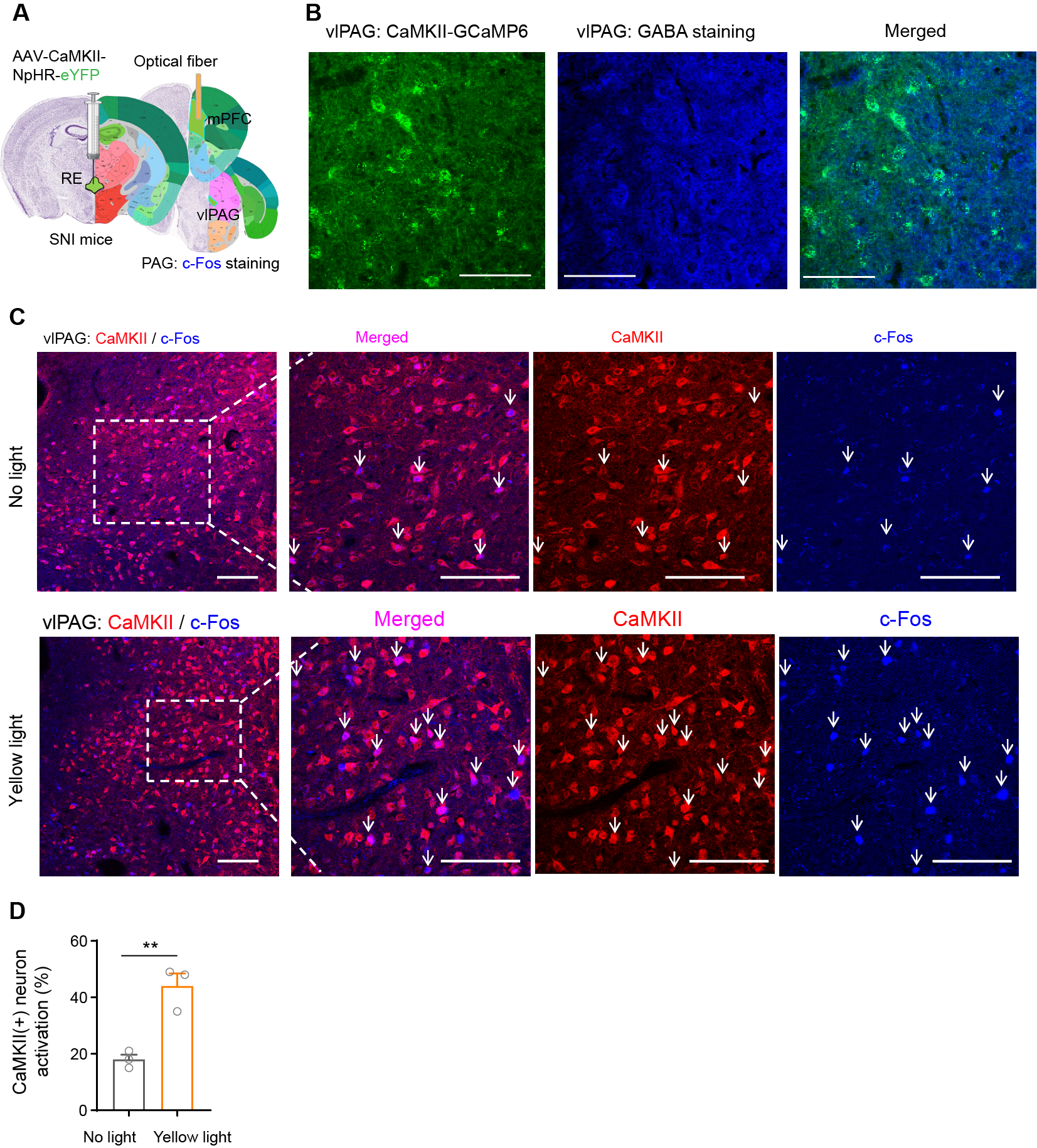

Supplement: S7 Fig — (A) Schematic diagram and representative image of viral expression in mPFC for optogenetic inhibition of RE-mPFC projections in SNI female mice. Nissl (left part of slices) and anatomical annotations (right part of slices) from the Allen Mouse Brain Atlas (https://mouse.brain-map.org) and Allen Reference Atlas-Mouse Brain (https://atlas.brain-map.org). (B, C) Effect of NpHR-mediated inhibition of RE-mPFC projections on mechanical PWT (F(2, 21) = 38.93, P < 0.0001) and thermal PWL (F(2, 21) = 40.05, P < 0.0001) in SNI mice (n = 8 mice). (D, E) Example heat maps (D) and quantification of time spent (E, Time, F(1, 14) = 4.15, P = 0.061) in the yellow-light-paired chamber during the preconditioning (Pre) and test sessions for NpHR mice (n = 8) and eYFP mice (n = 8). (F) Velocity of NpHR mice and eYFP mice in light-paired chamber during the test session (t = 0.73, P = 0.48, n = 8). (G–I) Raster plot showing sniffing episode (G, H) and total time spent sniffing (I, t = 4.35, P = 0.0007) in NpHR (n = 8) and eYFP (n = 8) mice tested during yellow light illumination of the RE-mPFC projection. (J, K) Immobility time in the FST (J, t = 2.77, P = 0.014) and TST (K, t = 4.46, P = 0.0005) in NpHR (n = 8) and eYFP (n = 8) mice during yellow light illumination of the RE-mPFC projection. Scale bars: 100 μm. *P < 0.05, **P < 0.01, ns not significant. One-way repeated measures ANOVAs with Tukey’s post-hoc analysis for (B, C). Two-way ANOVA with Tukey’s post-hoc analysis for (E). Two-tailed unpaired t-tests for (F, I − K). Data are available in S1 Data as a part of Supporting information. (TIFF) [file pbio.3003170.s007.tiff]

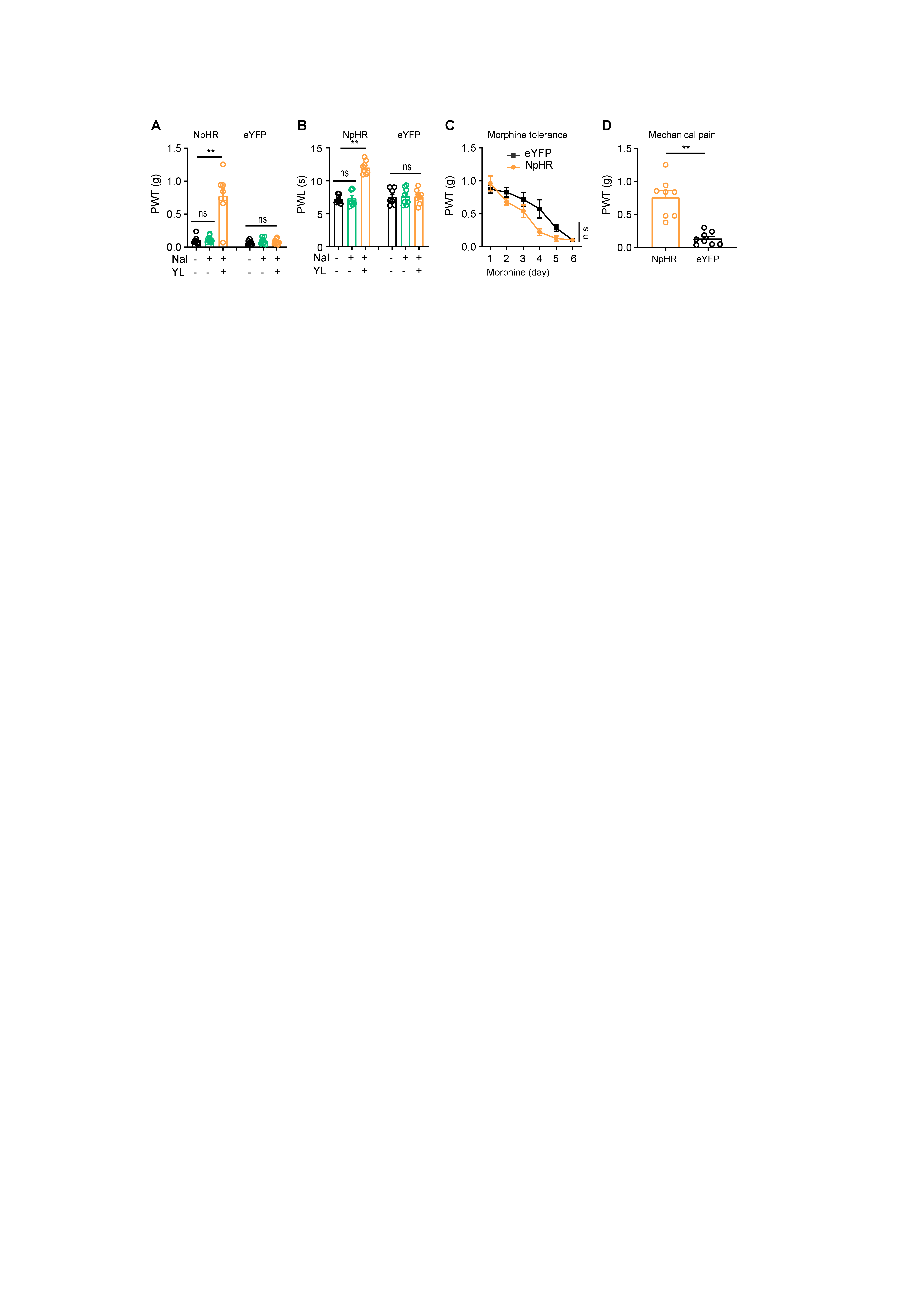

Supplement: S9 Fig — (A, B) The effect of naloxone on mechanical PWT and PWT before and during yellow light (YL) stimulation of the RE-mPFC projection in SNI mice. F(1, 14) = 36.52, P < 0.0001 for (A); F(1, 14) = 14.42, P = 0.002 for (B). n = 8 in each group. (C) NpHR and eYFP SNI mice develop significant antinociceptive tolerance to repetitive administration of 10 mg/kg morphine (i.p. twice daily for 6 days). NpHR vs. eYFP: F(5, 70) = 2.24, P = 0.06; Mor: i(3.14, 43.91) = 38.95, P < 0.0001; n = 8 in each group. (D) The effect of yellow light on mechanical PWT on day 7, 30 min after morphine administration in SNI mice. t = 5.72, P < 0.0001, n = 8 in each group. **P < 0.01; ns not significant. Two-way repeated measures ANOVAs with Tukey’s post-hoc analysis for (A − C); two-tailed unpaired t test for (D). Data are available in S1 Data as a part of Supporting information. (TIF) [file pbio.3003170.s009.tif]

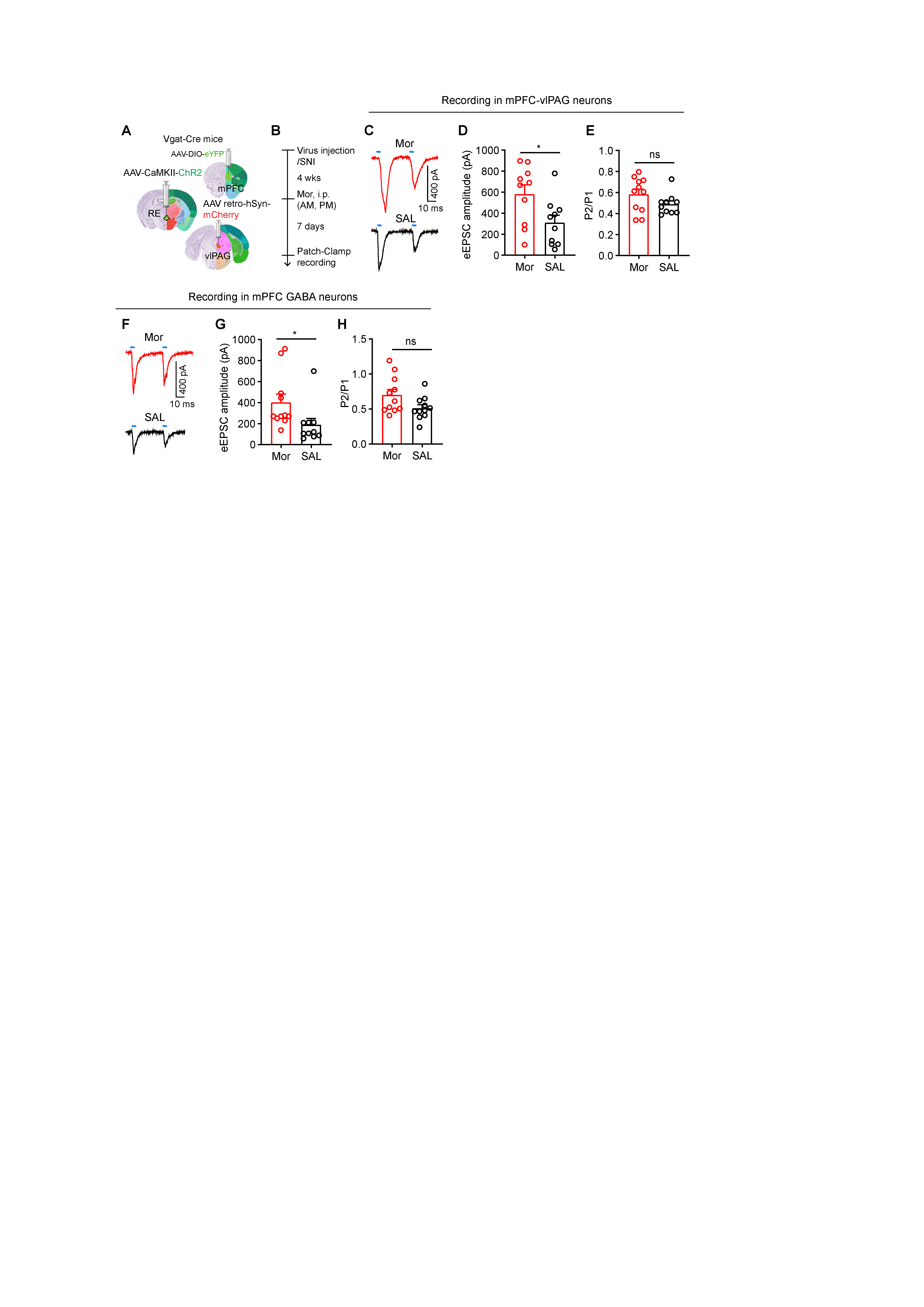

Supplement: S10 Fig — (A) Diagram showing virus injection strategy for labeling of mPFC GABA neurons and mPFC-vlPAG projection neurons and patch-clamp recording of their response to optogenetic activation of RE neurons. Nissl (left part of slices) and anatomical annotations (right part of slices) from the Allen Mouse Brain Atlas (https://mouse.brain-map.org) and Allen Reference Atlas-Mouse Brain (https://atlas.brain-map.org). (B) Experimental timeline. (C − E) Representative traces (C) and summary of eEPSC amplitude (D, t = 2.39, P = 0.028) and PPR (E, t = 1.56, P = 0.13)) recorded from mPFC-vlPAG neurons in response to 20 Hz blue light stimuli (paired pulses) in SNI mice. n = 10 cells from 4 mice each group. (F − H) Representative traces (F) and summary of eEPSC amplitude (G, t = 2.15, P = 0.045) and PPR (H, t = 2.03, P = 0.056) recorded from mPFC GABA neurons in response to 20 Hz blue light stimuli (paired pulses) in SNI mice. n = 10 cells from 4 mice each group. *P < 0.05, ns not significant. Two-tailed unpaired t-tests for (D, E, G, H). Data are available in S1 Data as a part of Supporting information. (TIF) [file pbio.3003170.s010.tif]
