# Supplementary material for: Excitatory projections from the nucleus reuniens to the medial prefrontal cortex modulate pain and depression-like behaviors in mice
Source: PLoS Biol. 2025 May 20;23(5):e3003170. doi: 10.1371/journal.pbio.3003170 (PMC12091829; doi:10.1371/journal.pbio.3003170)
Supplement: Attachment — Submitted filename: Responses to reviewers.docx [file pbio.3003170.s014.docx]

**REVIEW Comments**

**Reviewer #1:**

*Using a technical tour de force that includes selective expression of excitatory and inhibitory opsins, rabies-virus based anatomical tracing, in vivo calcium imaging and numerous behavioral tests, the authors establish a role of the thalamic nucleus reunines (RE) in both the sensory and cognitive components of neuropathic pain.*

*They show that RE neurons respond to mechanical pain stimuli, but not to simple touch, and that Ca signals in RE neurons increases when mice enter the open arms, face an unfamiliar mouse or receive an aversive air puff. They further show that optogenetic activation of RE neurons induces allodynia and aversion in naïve mice, while inhibition of RE neurons alleviates neuropathic pain symptoms in SNI mice. Additionally, the authors demonstrate that the (presumed glutamatergic) RE neurons provide monosynaptic excitatory inputs as well as polysynaptic inhibitory inputs to mPFC neurons that project to the vlPAG. They further analyzed the electrophysiological properties of mPFC-vlPAG neurons in SNI mice and claim that the I:E ratio is increased in these mice compared with sham-operated controls, and that firing of these mPFC neurons from SNI mice cannot reach the same firing frequency as the controls. Finally, the authors probed the behavioral effects of selective activation of the RE inputs to the mPFC. They found that in naïve mice, unilateral optogenetic activation of these inputs causes significant proalgesic effects on the ipsilateral paw, with no effect on the contralateral one. They then proceeded to show that in SNI mice optogenetic inhibition of the RE-mPFC pathway ameliorates both the cognitive/emotional and sensory neuropathic pain symptoms. Finally, they probed the effects of RE-mPFC circuit modulation of the calcium responses of vlPAG neurons elicited by tactile or thermal stimulation and found that inhibition of the RE-PFC pathways potentiates the responses of PAG neurons (ipsilaterally). A final set of experiments show that chemogenetic inhibition of the RE-pPFC pathway potentiates the analgesic response to submaximal doses of morphine. Importantly, the analgesic effect was detectable even in SNI mice with morphine tolerance.*

*Overall, the results are potentially very interesting, and the multi-pronged technical approach is remarkable. The paper however is not always rigorous, and some conclusions are not fully supported by the data.*

**Major concerns:**

*1. It is unclear how the PWT in SNI mice is ~ 0.01 g in control (off) condition in Fig. 3 E, and then goes to ~0.02 with light on. Yet in Supp. Fig 3, the control value is already higher than 0.02.*

**Response**: We thank the reviewer for this question. The mice we tested in Fig. 3E and those in Supp. Fig. 3 were from different cohorts. The differences in baseline PWT may result from biological variability among mice. We conducted two-tailed *t*-test to compare the baseline PWT between SNI mice in Fig. 3E and Supp. Fig. 3, but found no statistical significance. Using two-tailed paired *t*-test to compare PWT before and after treatment (e.g., light off vs. on in Fig. 3E) may eliminate the influence of variability in baseline PWT among mice on the effect of treatment.

*2. T**he authors used CamKII promoter to target glutamatergic neurons. Selectivity of this approach should be verified by performing GABA immunostaining because in the cortex CamKII expression is not completely selective for glutamatergic neurons (Veres at a. 2023)*

**Response**: We agree with the reviewer that CaMKII expression in the cortex is not completely selective for glutamatergic neurons. Since the RE is a nucleus composed exclusively of glutamatergic neurons (Learn Mem. 2019 Jul;26(7):191–205. doi: 10.1101/lm.048389.118), we used a CaMKII promoter-driven virus for selective modulation of RE glutamatergic neurons. In the vlPAG, we used the CaMKII promoter to drive GCaMP6 expression in vlPAG glutamatergic neurons. This specificity of CaMKII promoter was verified by immunostaining with a GABA antibody. We did not observe GCaMP6 in GABA(+) neurons (S8 Fig D).

*3. Figure 2K; contrary to what reported in the results, the ChR2 mice do not seem to show any preference on test day.*

**Response**: We appreciate the reviewer for this comment. We updated the example heatmap in Figure 2K to better reflect the data.

*4. Fig 4G. The peak of the inhibitory response is higher in SNI compared with sham and it is concluded that "I:E ratios in mPFC-vlPAG neurons in SNI mice were significantly greater…". However, the decay of the inhibitory current seems faster. Is the charge transfer larger in cells from SNI compared with sham mice? If not, it is very difficult to state that inhibition is increased. In general, I believe that it is not correct to compare the strength of synaptic inputs only on the basis of the current peak. The charge transfer must be considered, at least changes in the current time course cannot be excluded.*

**Response**: We agree with the reviewer in this point. To solve this puzzle, we calculated the peak amplitudes and the area under the curve (AUC) of blue light stimulation-evoked eEPSCs and eIPSCs in mPFC-vlPAG neurons. We found that the I:E ratios calculated using both the peak amplitudes and the AUC of eIPSCs and eEPSCs were significantly greater in mPFC-vlPAG neurons from SNI mice compared to those from sham controls. These data support that GABAergic inhibition onto these neurons is enhanced relative to excitatory drive following SNI (Fig. 4).

*5. The authors assume that cells lacking a voltage sag are interneurons; this however is not a suitable criterion to define interneurons in the mPFC, as commissural projecting pyramidal cells also do not have sag (in both rats and mice; see Dembrow and Johnston 2010; Avesar and Gulledge 2012; Leyre-Jackson and Thomas 2019). Thus, to claim that these recordings were performed from "electrophysiologically identified interneurons" (lines 311-12) is not correct.*

**Response**: We acknowledge the reviewer for this pioint. To address this concern, we changed methods to identify GABA neurons in revision. Similar to previous version, we used retrograde AAV to label vlPAG-projecting mPFC neurons, as shown in Figure 4B. In revision, we injected AAV-EF1a-DIO-eYFP into the mPFC in Vgat-Cre mice to label mPFC GABAergic neurons, and performed patch-clamp recording from these neurons. This approach allowed us to identified GABAergic interneurons more reliably.

*6. Fig 4K-M were these recordings performed in the presence or in the absence of synaptic blockers?*

**Response**: In Figure 4, patch-clamp recordings were performed without synaptic blockers.

*7. In the methods (Line 737) it is reported that EPSCs were recorded at -80 mV; in the Figure legend (Line 275) it is reported that they were recorded at -70 mV. Which is true?*

**Response**: We thank the reviewer for this point. All recordings of EPSCs were conducted at a holding potential of -70 mV. We corrected this mistake.

*8. The conclusion (Lines 335-38) that "Therefore, i**n SNI mice, the monosynaptic excitatory effect of the RE-mPFC projection onto mPFC vlPAG neurons may be compromised by enhancement of that onto mPFC GABAergic interneurons innervating mPFC-vlPAG neurons" is not sufficiently supported by the data. The change in maximum firing frequency may be due to intrinsic properties of the cells, not to altered synaptic input. This is easily tested by assessing the I/O curve in the presence of blockers of fast synaptic transmission.*

**Response**: We have added the experiment shown in S Figure 5. In the presence of synaptic blockers, the I/O curves in mPFC-vlPAG neurons in control and SNI mice became similar. This result suggests that the difference in firing frequency observed without synaptic blockers was predominantly due to changes in synaptic input from the RE-mPFC projection or other sources.

*9. One important experiment that is missing and, in my opinion, would greatly improve the significance of this paper concerns the potential effects of naloxone on the RE-mPFC pathway dependent analgesia.*

**Responses**: We have added the experiment shown in S figure 9A-B. “we tested the effect of optogenetic inhibition of the RE-mPFC projection in the presence of naloxone. Naloxone administration (2 mg/kg, *ip*) had no effect on the mechanical PWT or thermal PWL in SNI mice and did not block the analgesic effect of optogenetic inhibition of the RE-mPFC projection (S9 Fig A and B). These data indicate that the pain-relieving effect of RE-mPFC projection inhibition is not mediated by endogenous opioid signaling.”

**Minor points**

*1. The timing of each experiment (E.G. FST, TST) relative to SNI/Sham surgery is unclear. Were all tests performed 4 weeks after surgery?*

**Response**: We thank the reviewer for point out the missing information. We assessed these behaviors 4-5 weeks after SNI surgery. We added the information in the Methods section.

*2. Page 10: It is unclear how animals that have hyperalgesia showed "deficits in pain sensation"*

*Line 498-500 "Inhibiting the RE-mPFC Glu pathway amplified the pain responses…and ameliorated hyperalgesia" This sentence is self-contradictory*

**Response**: We thank the reviewer for these critiques. We changed the sentence in Page10 to “These results suggest that SNI mice exhibit hypersensitivity in pain sensation and display depression-like behaviors.”.

The sentence in Line 498-500 was changed to “Inhibiting the RE-mPFC Glu pathway increased the activity in vlPAG Glu neurons, and ameliorated hyperalgesia and depression-like behaviors in neuropathic pain mice.”

**Reviewer #2**

*This is a nice paper which extends our understanding of brain circuits involved in neuropathic pain states. Nice optogenetics and behavioral measurements were done. Well written, overall well done.*

**Comments:**

*1. One shortcoming is that only male mice were used in this study. The authors should perform one or two key sets of experiments in female mice*

**Response**: We admit that this is a major limitation of this study and we conducted experiments as the reviewer suggests. We performed optogenetic inhibition of RE neurons and the RE-mPFC projection in female SNI mice and observed analgesic effects similarly to that in male mice. We included the data in S3 Fig G-P and S7 Fig.

*2. Line 215: "In ChR2 mice subjected to SNI surgery, optogenetic activation of RE Glu neurons did not further decrease mechanical and thermal hypersensitivity (S3 Fig A, B)". Do you mean "increase hypersensitivity"? Also, this sentence should be moved until after the data on the inhibitory opsin are described. Maybe at line 245*

**Response**: We thank the reviewer for pointing out this mistake in wording. We changed this sentence into “In ChR2 mice subjected to SNI surgery, optogenetic activation of RE Glu neurons did not further reduce pain thresholds, as mechanical and thermal hypersensitivity remained unchanged (S3 Fig A, B).

*3. Line 533 - I do not think that there is a contradiction at all. It makes perfect sense that id there is feed forward inhibition in the mPFC, that stimulating inputs from the RE region would lead to less output from the mPFC and thus less excitability in the vlPAG. In fact, the authors already cite papers by Zhang et al (Cell Rep) and Huang et al (Nat Neurosci) - these papers parallel what the authors report here (except for BLA rather than RE inputs). It might be useful to more explicitly discuss the findings in the Huang study (ref 8) vis a vis what the authors observed in their current study - the mechanism sees quite similar especially when it comes to the synaptic processing in the mPFC GABA and pyramidal cells!*

**Response**: We thank the reviewer for this suggestion. In the Discussion session, we wrote: “Moreover, our findings extend the framework proposed by Huang et al. [8] to a new projection, the RE-mPFC pathway, and suggest that feedforward inhibition in the mPFC serves as a common mechanism for gating pyramidal neuron output to regulate distinct behavioral paradigms, such as chronic pain and emotional responses. Both the RE-mPFC projection investigated in our study and the RE-BLA projection in Huang et al. [8] converge on a key mechanism: increased excitatory inputs to the mPFC does not directly activate pyramidal neurons but instead amplify inhibitory control, thereby suppressing mPFC output, such as its regulation of the vlPAG in pain processing.”

*4. Methods:* *Viral injections were presumably done on the right hemisphere? If so, please state.*

*Merthods: SNI surgery - which side? Left?*

**Response**: We have added the information as the reviewer suggests. In the Methods section, viral injections were performed on the right hemisphere and the SNI surgery was performed on the same side as the viral injection (right side).

**Reviewer #3:**

*In this manuscript, Bao et al. investigates the role of the nucleus reuniens (RE) and its projections to the medial prefrontal cortex (mPFC) in modulating neuropathic pain and depression-like behaviors. The authors reveal that RE neurons are activated in response to pain-, anxiety-, and aversion-like stimuli, and the activation is more pronounced in a neuropathic pain state. Activation of the RE-mPFC pathway induces hyperalgesia and depression-like behaviors, while inhibition of this pathway alleviates these behaviors in neuropathic pain mice. Mechanistically, in neuropathic pain, the RE-mPFC pathway preferentially innervates local GABAergic circuits in the mPFC, reducing activity in mPFC-vlPAG neurons. These findings highlight the RE-mPFC pathway as a critical circuit in neuropathic pain and its comorbid depression, and a potential therapeutic target for non-opioid treatments. Overall, in this study, the experiments are reasonably designed, data are solid to support the conclusions, and results are appropriately interpreted. The authors should address my concerns to improve this manuscript.*

**Main points:**

*1. In Figure 1I-O, the authors only show the responses of RE neurons to anxiety, social stimulus, and air puff in SNI mice. They should examine these responses in naïve mice. The comparison of the responses between naïve and SNI mice could address whether RE neurons exhibit hyperexcitability and respond differently to these stimuli in SNI mice*

**Response**:  We included the responses of RE neurons to anxiety, social stimuli, and air puff in naïve mice in Figure 1I-T. We observed that RE neurons in SNI mice enhanced activity more strongly than those in naïve mice in response to anxiety and air puff, but similarly to naïve mice in response to social interaction.

*2. In Figure 2K, L, the authors should examine movement velocity in two compartments of the CPP chamber during precondition and test sessions. Similar changes should be made in Figure 3 and 5.*

**Response**: We thank the reviewer for this suggestion. We analyzed the video file and found that movement velocity in two compartments of the CPP chamber remained similar between precondition and test sessions. We included these data in Figures 2M, 3k, 5L and 5U.

*3. Figure 4 shows that in mPFC-vlPAG neurons, stimulation of the RE-mPFC glutamatergic projection evoked not only direct EPSCs, but also indirect IPSCs mediated by mFPC GABAergic interneurons. The optogenetic stimulation of the RE-mPFC projection causes less firing in mPFC-vlPAG neurons in SNI mice than sham mice. The authors explained that this phenomenon may be a result of the enhanced indirect IPSCs on mPFC-vlPAG neurons. To confirm this hypothesis, the authors should examine whether the difference in photo-stimulation of the RE-mPFC projection-induced firing between sham and SNI mice is eliminated by inhibition of GABAergic synaptic transmission.*

**Response**: We thank the reviewer for this suggestion. We added the experiment shown in S Figure 5. Application of GABA blockers (10 μM BIC) abolished the difference in firing frequency during blue light stimulation of ChR2-expressing RE terminals (S5 Figure G-H). This indicates that the differences in firing frequency observed without synaptic blockers were predominantly due to changes in synaptic input from the RE-mPFC projection.

*4. Figure 6 shows that inhibition of the RE-mPFC projection enhanced pain responses in vlPAG neurons* *upon stimulation on the ipsilateral hind paw but not that on the contralateral hind paw. The authors should discuss this interesting result.*

**Response**: We included a discussion of this result in the Discussion section. We wrote “We demonstrated that inhibition of the RE-mPFC pathway enhanced pain responses of vlPAG Glu neurons to stimulation of the ipsilateral hind paw in neuropathic pain mice. This finding aligns with the known lateralization of descending pain modulatory pathways, where the vlPAG primarily operates in an ipsilateral manner [8, 14]. In the context of neuropathic pain, this ipsilateral-specific enhancement of vlPAG Glu responses upon RE-mPFC inhibition could reflect circuit-level adaptations or sensitizations that reinforce pain processing on the injured side. These results highlight the role of lateralized dynamics within a tertiary neural circuit ( RE^Glu^→mPFC^Glu^/(mPFC^GABA^-mPFC^Glu^)→vlPAG^Glu^ neurons) in pain modulation.”.

*5. Figure 7 shows that inhibition of RE neurons projecting to the mPFC exerted analgesic effect in mice subjected to both acute and chronic morphine. The authors should examine whether naloxone can block this analgesic effect to confirm its non-opioid property. Additionally, the authors should clarify whether repetitive morphine administration modifies the RE-mPFC pathway. If this is the case, the role of this pathway will be extended from neuropathic pain to morphine tolerance.*

**Response**: We agree with the reviewer for these insightful suggestions. In S Figure 9, we show that naloxone did not block the analgesic effect of optogenetic inhibition of the RE-mPFC projection. In S Figure 10, we show that repetitive morphine administration enhances the RE-mPFC projection onto both vlPAG-projecting mPFC neurons and mPFC GABA neurons. Further investigations are needed to elucidate the role of the synaptic plasticity in morphine tolerance.

*6. S5 Figure shows that mPFC GABAergic interneurons receive innervations from the RE-mPFC projection. The authors should examine whether EPSCs on mPFC GABAergic interneurons induced by optogenetic stimulation of this projection are enhanced in SNI mice. The result can provide further evidence to elaborate the RE-mPFC circuit involved in neuropathic pain.*

**Response**: We thank the reviewer for this suggestion. We injected AAV-CaMKII-ChR2-eYFP into the RE and AAV-EF1a-DIO-eYFP in the mPFC in Vgat-Cre mice, and recorded light-evoked EPSCs from eYFP-labeled mPFC GABA neurons (S Figure 5A, 5B). We found that SNI enhanced the RE-mPFC glutamatergic projection onto mPFC GABA neurons (S Figure 5C-F).

**Minor points**

*1. In Abstract, the authors should clarify the sentence “the RE-mPFC projection was enhanced with a marked preference for the part innervating local GABAergic circuits to that controlling mPFC neurons projecting to the ventrolateral periaqueductal gray (vlPAG)” . The word “local” is confusing (it means in the RE or mPFC?). The authors should mention “GABAergic circuit in the mPFC”.*

**Response**: We meant that ‘local’ is mPFC. We corrected this sentence and wrote “the RE-mPFC projection was enhanced with a marked preference for the part innervating GABAergic circuits in the mPFC to that controlling mPFC neurons projecting to the ventrolateral periaqueductal gray (vlPAG).”.

*2. In Figure 3E-H, the authors should change “NpHR” and “eYFP” into “light”, and add an annotation (NpHR mice or eYFP mice) above each histogram.*

**Response**: We thank the reviewer for this correction. We modified Figure 3E-H.

*3. In line 540-542, “we did observe that optogenetic stimulation of the RE-mPFC projection evoked larger IPSC in SNI mice than in sham mice, resulting in a larger I/E ratio.” Should be changed into “we did observe that optogenetic stimulation of the RE-mPFC projection evoked larger IPSCs in mPFC-vlPAG neurons in SNI mice than in sham mice, resulting in a larger I/E ratio.”*

**Response**: We corrected this sentence.
